# Karyotype Reorganization in the Hokou Gecko (*Gekko hokouensis*, Gekkonidae): The Process of Microchromosome Disappearance in Gekkota

**DOI:** 10.1371/journal.pone.0134829

**Published:** 2015-08-04

**Authors:** Kornsorn Srikulnath, Yoshinobu Uno, Chizuko Nishida, Hidetoshi Ota, Yoichi Matsuda

**Affiliations:** 1 Laboratory of Animal Genetics, Department of Applied Molecular Biosciences, Graduate School of Bioagricultural Sciences, Nagoya University, Furo-cho, Chikusa-ku, Nagoya, Aichi, Japan; 2 Laboratory of Animal Cytogenetics and Comparative Genomics, Department of Genetics, Faculty of Science, Kasetsart University, 50 Ngamwongwan, Chatuchak, Bangkok, Thailand; 3 Center for Advanced Studies in Tropical Natural Resources, National Research University-Kasetsart University (CASTNAR, NRU-KU), Kasetsart University, Bangkok, Thailand; 4 Department of Natural History Sciences, Faculty of Science, Hokkaido University, Kita 10, Nishi 8, Kita-ku, Sapporo, Hokkaido, Japan; 5 Institute of Natural and Environmental Sciences, University of Hyogo, and Museum of Nature and Human Activities, Sanda, Hyogo, Japan; University of Florence, ITALY

## Abstract

The Hokou gecko (*Gekko hokouensis*: Gekkonidae, Gekkota, Squamata) has the chromosome number 2n = 38, with no microchromosomes. For molecular cytogenetic characterization of the gekkotan karyotype, we constructed a cytogenetic map for *G*. *hokouensis*, which retains the ancestral karyotype of Gekkota, with 86 functional genes, and compared it with cytogenetic maps for four Toxicofera species that have many microchromosomes (*Elaphe quadrivirgata*, *Varanus salvator macromaculatus*, *Leiolepis reevesii rubritaeniata*, and *Anolis carolinensis*) and that for a lacertid species (*Lacerta agilis*) with only one pair of autosomal microchromosomes. Ten pairs of *G*. *hokouensis* chromosomes [GHO1, 2, 3, Z(4), 6, 7, 8, 13, 14, and 15] showed highly conserved linkage homology with macrochromosomes and/or macrochromosome arms of the four Toxicofera species and corresponded to eight *L*. *agilis* macrochromosomes (LAG). However, GHO5, GHO9, GHO10, GHO11, and LAG6 were composed of chromosome segments that have a homology with Toxicofera microchromosomes, and no homology was found in the chromosomes between *G*. *hokouensis* and *L*. *agilis*. These results suggest that repeated fusions of microchromosomes may have occurred independently in each lineage of Gekkota and Lacertidae, leading to the disappearance of microchromosomes and appearance of small-sized macrochromosomes.

## Introduction

Karyotypes of non-avian reptiles have been extensively diversified [1] since Sauropsida (all existing reptiles and birds) diverged from Synapsida around 320 million years ago (MYA) [2]. Generally, turtles have many microchromosomes, which are designated by their chromosome morphologies as dot-shaped chromosomes whose centromere positions are undetectable [3, 4], whereas all crocodilian species lack microchromosomes. Crocodilians show low karyotypic variation with respect to both chromosome number (chromosome arm number in particular) and chromosome morphology [1, 5]. In squamate reptiles, both macrochromosomes and microchromosomes are commonly found in Scincoidea and Episquamata exclusive of Lacertidae; in contrast, only a few or no microchromosomes are found in Lacertidae and Gekkota [1, 6].

Until recently, not much information was available on the process of chromosomal reorganization, which causes extensive karyotypic variation in reptiles, such as appearance or disappearance of microchromosomes. However, recent comparative gene mapping of several non-avian reptile species (*Pelodiscus sinensis*, Testudines; *Crocodylus siamensis*, Crocodilia; *Lacerta agilis*, *Elaphe quadrivirgata*, *Varanus salvator macromaculatus*, *Leiolepis reevesii rubritaeniata*, *Pogona vitticeps*, and *Anolis carolinensis*, Episquamata) with the chicken revealed the extensive homology between avian and reptilian chromosomes and suggested that the common ancestor of amniotes may have had many microchromosomes, whose linkages have been conserved between the chicken and reptiles [7–16]. In squamate reptiles, however, microchromosomes, supposedly occurring in large number in the ancestral state, are considered to have reduced because of their fusions with macrochromosomes and/or with other microchromosomes [9–13, 15].

Lacertid lizards of the superfamily Lacertoidea are classified into Episquamata that generally have many microchromosomes [1, 6, 17]; nevertheless, karyotypes of lacertid lizards include few or no microchromosomes [1, 18–22]. Comparative mapping of 86 functional genes in *L*. *agilis* revealed that linkage groups of chromosomes are highly conserved between *L*. *agilis* and Toxicofera species (*V*. *salvator macromaculatus*, Anguimorpha; *L*. *reevesii rubritaeniata*, Iguania; and *E*. *quadrivirgata*, Serpentes), whose karyotypes consist of macrochromosomes and many indistinguishable microchromosomes [9–13]. This finding suggests that the karyotypes of lacertid lizards probably have resulted from repeated fusions of microchromosomes, leading to the scarcity or complete absence of these elements.

Gekkota, which includes seven families (Diplodactylidae, Carphodactylidae, Pigopodidae, Eublepharidae, Sphaerodactylidae, Gekkonidae, and Phyllodactylidae), is phylogenetically located at the base of squamate reptiles exclusive of the Dibamidae, being estimated to have diverged from the common ancestor of non-dibamid squamates around 170–240 MYA [1, 6, 23, 24]. This phylogenetic relationship suggests that geckos may retain the ancestral squamate karyotypes. Notwithstanding this, however, geckos actually have unique karyotypes that are also characterized by scarcity of microchromosomes with a very few exceptions [25], as observed in lacertid lizards. The karyotypes of geckos are highly diversified, ranging from 2n = 16 to 48, and the number of chromosome arms varies considerably (FN = 32 to 76) [1, 26, 27]. These data suggest that several fusions or fissions and multiple pericentric inversions occurred independently in each family and between species within the same family, leading us to suppose that microchromosomes in Episquamata and Scincoidea appeared by repeated breakages of macrochromosomes in the ancestral squamate karyotype [1, 6, 28–30]. However, karyotypes resembling those of Gekkota are not found in majority of the squamate reptiles, although Gekkota is phylogenetically located at the base of all squamates reptiles but Dibamidae; therefore, an alternative explanation should be considered: the microchromosomes actually disappeared by fusions between macrochromosomes and microchromosomes and/or between microchromosomes in the gekkotan lineage. Nevertheless, no evidence has been obtained to verify these possibilities. Comparative analysis of chromosomal syntenies between geckos and other squamate reptiles is therefore a good approach to delineate the process of karyotype evolution in squamate reptiles.

The Hokou gecko (*Gekko hokouensis*: Gekkonidae, Gekkota) is widely distributed in southeastern China; Taiwan; and the Ryukyu Islands and southern Kyushu, Japan [31–33]. The diploid chromosome number of *G*. *hokouensis* is 38 with no indistinguishable microchromosomes and it retains the ancestral karyotype of Gekkota [34–38], whereas there is a regional variation in sex chromosome constitution (homomorphic sex chromosomes and ZZ/ZW-type heteromorphic sex chromosomes) [36]. In our previous study, comparative mapping of *G*. *hokouensis* homologs of the chicken Z-linked genes [39] originally revealed that *G*. *hokouensis* and birds have the same origin for sex chromosomes, which are derived from the same autosomal pair of the common ancestor. However, the process of dramatic chromosomal reorganization in this species is still unknown because the homology of *G*. *hokouensis* autosomes with those of other squamate reptiles has not yet been studied. In this study, to characterize *G*. *hokouensis* chromosomes, we constructed a comparative cytogenetic map with 86 functional genes, 18S–28S and 5S ribosomal RNA (rRNA) genes, and telomeric TTAGGG repeats by using fluorescence *in situ* hybridization (FISH) and compared the chromosome homology of *G*. *hokouensis* with five Episquamata species (four Toxicofera species *E*. *quadrivirgata*, *V*. *salvator macromaculatus*, *L*. *reevesii rubritaeniata* and *A*. *carolinensis*, and one lacertid species *L*. *agilis*), as well as the chicken. Here, we have delineated the process of chromosomal reorganization in Gekkota and discussed karyotype evolution in squamate reptiles.

## Materials and Methods

### Specimen, cell culture, and chromosome preparation

Testes, which were collected from an adult male Hokou gecko (*G*. *hokouensis*) and frozen in our previous study [39], were used for RNA isolation. Fibroblasts from a female *G*. *hokouensis* used in our previous study [39] were recovered from liquid nitrogen and cultured. After thawing, the cells were cultured in Dulbecco's modified Eagle's medium (Life Technologies-Gibco, Carlsbad, CA, USA) supplemented with 15% fetal bovine serum (Life Technologies-Gibco), 100 μg/ml kanamycin, and 1% antibioticantimycotic (PSA) (Life Technologies-Gibco). The cultures were incubated at 26°C in a humidified atmosphere of 5% CO_2_ in air. Animal care and all experimental procedures were approved by the Animal Experiment Committee, Hokkaido University (approved no. 08–0214), and were conducted according to Regulations on Animal Experiments in Hokkaido University. For replication banding, fibroblasts in the logarithmic growth phase were incubated with 5-bromo-2'-deoxyuridine (12 μg/ml) (Sigma-Aldrich, St. Louis, MO, USA) for 12 h, including 45 min of colcemid treatment (120 ng/ml) (Nacalai Tesque, Kyoto, Japan), before harvest. The cells were harvested by treatment with trypsin, suspended in 0.075 M KCl at room temperature for 20 min, and fixed with methanol/ acetic acid (3:1) three times. The cell suspension was dropped on cleaned glass slides and air-dried. After staining the chromosome slides with Hoechst 33258 (1 μg/ml) for 5 min, the slides were heated at 65°C for 3 min and exposed to UV light at 65°C for an additional 6 min [40]. The slides were maintained at –80°C until use.

### Molecular cloning of cDNA fragments of functional genes

cDNA fragments of 18 functional genes (*FBXW11*, *BRD2*, *CACNB4*, *EEF2*, *HDAC3*, *SS18*, *EXOC1*, *RAP1GDS1*, *WAC*, *HSPA8*, *ATP2A2*, *SBNO1*, *MYST2*, *DYRK2*, *TTC26*, *SH3PXD2A*, *TLOC1*, and *TRIM37*), which have been mapped to *L*. *reevesii rubritaeniata* and *E*. *quadrivirgata* chromosomes [9–11], were cloned from a male *G*. *hokouensis* by using the PCR primers of our previous study [11, 13]. Testes of *G*. *hokouensis* were homogenized and lysed with TRIzol Reagent (Life Technologies, Carlsbad, CA, USA), and total RNA was extracted according to the manufacturer’s instructions. The cDNA fragments were obtained using RT-PCR with Oligo (dT)_12–18_ Primer and SuperScript II RNase H^−^Reverse Transcriptase (Life Technologies) and used as PCR templates to amplify *G*. *hokouensis* homologs. cDNA amplification was performed using 20 μl of 1× Ex Taq buffer that contained 1.5 mM MgCl_2_, 0.2 mM dNTPs, 5.0 μM degenerate primers, and 0.25 U of TaKaRa Ex Taq (TaKaRa Bio, Otsu, Japan). PCR conditions were as follows: an initial denaturation at 94°C for 2 min, followed by 35 cycles of 94°C for 30 s, 52°C for 30 s, and 72°C for 35 s, and a final extension at 72°C for 10 min. The PCR products were cloned using pGEM-T Easy Vector System I (Promega, Madison, WI, USA). Nucleotide sequences of the cDNA fragments were determined using an ABI 3130 Automated Capillary DNA Sequencer (Life Technologies-Applied Biosystems, Carlsbad, CA, USA). The nucleotide sequences were searched for homologies with those of the chicken and anole lizard (*A*. *carolinensis*) in the National Center for Biotechnology Information (NCBI) database to confirm if cDNA fragments of the objective genes were obtained exactly by using the blastx and blastn programs (http://blast.ncbi.nlm.nih.gov/Blast.cgi), and they were deposited in the DNA Data Bank of Japan (http://www.ddbj.nig.ac.jp/index-e.html).

### FISH mapping

Chromosomal locations of 80 functional genes, 18S–28S and 5S rRNA genes, and telomeric (TTAGGG)n sequences were determined using FISH, as described previously [40, 41]. For FISH mapping of functional genes, we used cDNA fragments of 18 genes cloned from *G*. *hokouensis* in the present study and cDNA fragments of 62 genes that were cloned from three other squamate reptiles of our previous studies: 30 genes from *L*. *agilis* [13], 31 genes from *L*. *reevesii rubritaeniata* [11], and one gene from *E*. *quadrivirgata* (Matsubara et al., unpublished data) (Table 1). For FISH mapping of 18S–28S and 5S rRNA genes and telomeric (TTAGGG)n sequences, we used a partial 1.8-kb genomic DNA fragment (pCSI1) of the 8.2-kb fragment of *C*. *siamensis* 18S–28S rRNA genes (EU727190), a 99-bp genomic DNA fragment of *C*. *siamensis* 5S rRNA genes (pCSI5S; EU723235), and biotin-labeled 42-bp TTAGGG repeat, respectively. We labeled 250 ng of DNA fragments with biotin-16-dUTP (Roche Diagnostics, Basel, Switzerland) by nick translation, according to the manufacturer’s protocol. After hybridization of biotin-labeled cDNA fragments to *G*. *hokouensis* chromosomes, the probes were incubated with goat anti-biotin antibody (Vector Laboratories, Burlingame, CA, USA) and stained with Alexa Fluor 488 rabbit anti-goat IgG (H + L) conjugate (Life Technologies-Molecular Probes). Slides were subsequently stained with 0.75 μg/ml propidium iodide.

**Table 1:** List of 86 cDNA clones mapped to the Hokou gecko (*Gekko hokouensis*) chromosomes and their chromosomal locations in the sand lizard (*Lacerta agilis*), the water monitor lizard (*Varanus salvator macromaculatus*), the butterfly lizard (*Leiolepis reevesii rubritaeniata*), the Japanese four-striped rat snake (*Elaphe quadrivirgata*), the green anole (*Anolis carolinensis*), and the chicken (*Gallus gallus*).

Gene symbol	Origin of cDNA fragment	Sequenced length of cDNA fragment (bp)	Chromosomal location							
			*G*. *hokouensis*	*L*. *agilis*	*V*. *salvator macromaculatus*	*L*. *reevesii rubritaeniata*	*E*. *quadrivirgata*	*A*. *carolinensis*	*G*. *gallus*	Accession number
*XAB1*	*L*. *reevesii rubritaeniata*	489	1p	3	2p	1p22.4	1p	^___^	3	AB490344
*FBXW11*	*G*. *hokouensis*	926	1p	3	2p	^___^	1p	1	13	AB792691
*ESR1*	*L*. *reevesii rubritaeniata*	951	1p	3	2p	1p21.2	1p	^___^	3	AB490345
*SOX9* ^a^	*L*. *reevesii rubritaeniata*	603, 717	1q	2	1q	2q11.2 –q11.4	2q	^___^	18	AB490350, AB490351
*TOB1*	*L*. *agilis*	950	1q	2	1q	^___^	^___^	2	18	AB794087
*RUFY1*	*L*. *reevesii rubritaeniata*	545	1q	2	1q	2q12.2 –q21.1	2q	2	13	AB490352
*BRD2*	*G*. *hokouensis*	732	1q	2	1q	2q22.2	^___^	^___^	16	AB792685
*TKT*	*L*. *reevesii rubritaeniata*	943	1q	2	1q	2q11.1	2q	2	12	AB490349
*ALAS1*	*L*. *agilis*	1,060	1q	2	1q	^___^	^___^	2	12	AB794074
*ACVR1*	*L*. *agilis*	845	2p	1	2p	^___^	^___^	^___^	7	AB794073
*CACNB4* ^a^	*G*. *hokouensis*	1,008, 1,201	2p	1	2q	1q12.2 –q13.1	1q	^___^	7	AB792686, 792687
*WT1*	*L*. *reevesii rubritaeniata*	542	2p	1	2q	1q21.1 –q22.1	1q	1	5	AB490347
*DYNC1H1*	*L*. *reevesii rubritaeniata*	997	2q	1	2q	1q32.1 –q32.3	1q	^___^	5	AB490348
*CYP2C21-like*	*L*. *agilis*	1,331	2q	1	2q	^___^	^___^	^___^	^___^	AB794068
*EEF2*	*G*. *hokouensis*	1,037	3	19	Micro	Micro	1q	1	28	AB792689
*ZNF326*	*L*. *reevesii rubritaeniata*	892	3	7	8p	4q12.1 –q12.3	3q	4	8	AB490366
*RPE65*	*L*. *agilis*	1,130	3	7	8p	^___^	^___^	^___^	8	AB793733
*USP49*	*L*. *agilis*	1,210	3	7	8q	^___^	^___^	4	26	AB794088
*CNTN2*	*L*. *agilis*	922	3	7	8q	^___^	^___^	4	26	AB793728
*RBM12*	*L*. *reevesii rubritaeniata*	943	3	7	8q	4q21.2 –q22.1	3q	4	20	AB490367
*RPN2*	*L*. *agilis*	1,229	3	7	8q	^___^	^___^	^___^	20	AB794084
*ACO1/IREBP* ^b^	*G*. *hokouensis*	1,122	4 (ZW)	11–18	1p	2p11.2 –p11.4	2p	^___^	Zq	AB326219, AB326220
*RPS6* ^b^	*G*. *hokouensis*	593	4 (ZW)	11–18	1p	2p11.3 –p12	2p	2	Zp	AB326221
*DMRT1* ^b^	*G*. *hokouensis*	637	4 (ZW)	11–18	1p	2p12 –p21	2p	2	Zp	AB326222
*CHD1* ^b^	*G*. *hokouensis*	1,263	4 (ZW)	11–18	1p	2p21	2p	2	Zq	AB326217, AB326218
*GHR* ^b^	*G*. *hokouensis*	852	4 (ZW)	11–18	1p	2p22 –p23.3	2p	2	Zp	AB326214
*ATP5A1* ^b^	*G*. *hokouensis*	990	4 (ZW)	11–18	1p	2p23.1 –p23.3	2p	1	Zp	AB326215, AB326216
*TMEM57*	*L*. *agilis*	800	5	6	Micro	^___^	^___^	^___^	23	AB794086
*RNF19B*	*L*. *agilis*	942	5	6	Micro	^___^	^___^	^___^	23	AB793732
*CUL4B*	*L*. *reevesii rubritaeniata*	723	5	11–18	Micro	Micro	^___^	^___^	4p	AB490387
*ATRX*	*L*. *reevesii rubritaeniata*	804	5	11–18	Micro	Micro	Micro	^___^	4p	AB490386
*AR*	*L*. *reevesii rubritaeniata*	941	5	11–18	Micro	Micro	Micro	^___^	4p	AB490385
*CHD2* ^a^ ^,^	*L*. *reevesii rubritaeniata*	654, 692	6q	11–18	Micro	Micro	^___^	^___^	10	AB490388, AB490389
*HDAC3*	*G*. *hokouensis*	976	6q	8	7p	4p11.1 –p11.2	3p	4	13	AB792692
*SS18*	*G*. *hokouensis*	881	6q	8	7q	4p12.2 –p21.2	3p	4	2q	AB792697
*ENPP2*	*L*. *reevesii rubritaeniata*	961	6q	8	7q	4p22.2 –p22.4	3p	^___^	2q	AB490363
*POLG*	*L*. *agilis*	1,565	6q	11–18	Micro	^___^	Micro	^___^	10	AB794083
*UCHL1*	*L*. *reevesii rubritaeniata*	595	7	10	3p	5p11.1 –p12.1	6p	5	4q	AB490372
*EXOC1*	*G*. *hokouensis*	1,171	7	10	3q	5p11.2 –p12.2	7p	5	4q	AB792690
*ACSL1*	*L*. *reevesii rubritaeniata*	748	7	10	3q	5p12.1 –p12.2	7q	5	4q	AB490370
*DCLK2*	*L*. *reevesii rubritaeniata*	688	7	10	3q	5p12.1 –p21	^___^	5	4q	AB490369
*SMAD1*	*L*. *agilis*	944	7	10	3q	^___^	^___^	5	4q	AB794085
*RAP1GDS1*	*G*. *hokouensis*	1,044	7	10	3q	5p21 –p22.2	7q	^___^	4q	AB792702
*CTNNB1*	*L*. *reevesii rubritaeniata*	1,201	8	11–18	4q	6q11	Zp	6	2p	AB490379
*TOP2B*	*L*. *agilis*	1,639	8	11–18	4q	^___^	^___^	^___^	2p	AB793737
*WAC*	*G*. *hokouensis*	858	8	11–18	4q	6q21 –q23	Zp	6	2p	AB792701
*GAD2*	*L*. *reevesii rubritaeniata*	672	8	11–18	4q	6q21 –q23	Zp	6	2p	AB490380
*ARNT*	*L*. *agilis*	1,034	9	11–18	Micro	^___^	^___^	^___^	25	AB794075
*ENO1*	*L*. *agilis*	917	9	6	Micro	^___^	Micro	^___^	21	AB794078
*DNM1*	*L*. *agilis*	1,014	9	6	Micro	^___^	Micro	^___^	17	AB794076
*PPP2R1A*	*L*. *agilis*	1,169	9	6	Micro	^___^	^___^	^___^	^___^	AB793731
*GRIN1*	*L*. *agilis*	893	9	6	Micro	^___^	Micro	^___^	17	AB794080
*EEF2K*	*L*. *agilis*	970	10	11–18	Micro	^___^	Micro	^___^	14	AB794077
*UBN1*	*L*. *agilis*	1,153	10	11–18	Micro	^___^	^___^	^___^	14	AB794072
*PDXDC1*	*L*. *agilis*	1,619	10	11–18	Micro	^___^	^___^	^___^	14	AB794082
*HSPA8*	*G*. *hokouensis*	952	10	11–18	Micro	Micro	Micro	Micro	24	AB792693
*NF2*	*L*. *reevesii rubritaeniata*	940	11	11–18	Micro	Micro	^___^	^___^	15	AB490393
*SF3A1*	*L*. *reevesii rubritaeniata*	937	11	11–18	Micro	Micro	^___^	^___^	15	AB490394
*ATP2A2*	*G*. *hokouensis*	1,023	11	11–18	Micro	Micro	Micro	Micro	15	AB792684
*SBNO1*	*G*. *hokouensis*	1,050	11	11–18	Micro	Micro	^___^	Micro	15	AB792695
*MYST2*	*G*. *hokouensis*	1,315	12	11–18	4p	6p21.1 –p22.2	Zq	6	27	AB792694
*STAT3*	*L*. *agilis*	1,654	12	11–18	4p	^___^	^___^	6	27	AB793734
*TOP2A* ^a^	*L*. *agilis*	882, 477	12	11–18	4p	^___^	^___^	6	27	AB793735, AB793736
*TPT1*	*L*. *reevesii rubritaeniata*	438	13q	4	5q	3p11.1 –q11	4p	^___^	1q	AB490359
*IPO5* ^a^	*L*. *agilis*	703, 492	13q	4	5q	^___^	^___^	3	1q	AB793729, AB793730
*EIF2S3*	*L*. *reevesii rubritaeniata*	733	13q	4	5q	3q12.3 –q21.1	4p	3	1q	AB490361
*OCA2*	*L*. *reevesii rubritaeniata*	782	13q	4	5q	3q12.1 –q12.2	^___^	3	1q	AB490360
*BRD7*	*L*. *reevesii rubritaeniata*	784	13q	11–18	Micro	Micro	Micro	^___^	11	AB490390
*ELMOD1*	*L*. *reevesii rubritaeniata*	682	13q	4	5q	3q22.1 –q22.3	4q	^___^	1q	AB490362
*ACTN4*	*L*. *reevesii rubritaeniata*	1,069	14p	11–18	Micro	Micro	^___^	^___^	^___^	AB490396
*DYRK2*	*G*. *hokouensis*	1,011	14q	9	3p	5q12 –q21.2	^___^	^___^	1p	AB792688
*RANGAP1*	*L*. *reevesii rubritaeniata*	1024	14q	9	3p	5q21.2 –q22.1	6q	5	1p	AB490374
*TTC26*	*G*. *hokouensis*	744	14q	9	3p	5q21.3 –q22.3	6q	^___^	1p	AB792700
*SOX5* ^a^	*L*. *reevesii rubritaeniata*	851, 705	14q	9	3p	5q22.1 –q22.4	^___^	5	1p	AB490376, AB490377
*ADAM12*	*L*. *agilis*	933	15q	5 (Z)	6q	^___^	^___^	3	6	AB794067
*PSAP*	*L*. *reevesii rubritaeniata*	1,325	15q	5 (Z)	6q	3p11.2 –p12.2	5q	^___^	6	AB490358
*BTRC*	*L*. *reevesii rubritaeniata*	889	15q	5 (Z)	6q	3p21.1 –p21.2	^___^	^___^	6	AB490357
*SH3PXD2A*	*G*. *hokouensis*	1,235	15q	5 (Z)	6q	3p22.1	5q	3	6	AB792696
*SLIT1*	*L*. *agilis*	995	15q	5 (Z)	6q	^___^	^___^	^___^	6	AB794071
*SKIL*	*L*. *agilis*	1,686	15q	5 (Z)	6q	^___^	^___^	3	9	AB794070
*EPHA4*	*L*. *agilis*	888	15q	5 (Z)	6q	^___^	^___^	3	9	AB794079
*TLOC1*	*G*. *hokouensis*	836	15q	5 (Z)	6q	3p22.3	5q	3	9	AB792698
*NCL*	*L*. *agilis*	1,780	15q	5 (Z)	6q	^___^	^___^	^___^	9	AB794069
*KRT8*	*L*. *agilis*	835	16q	2	Micro	^___^	^___^	^___^	^___^	AB794081
*TRIM37*	*G*. *hokouensis*	1,186	17q	1	Micro	Micro	1p	^___^	19	AB792699
*AMH* ^a^	*E*. *quadrivirgata*	712, 709	17q	1	Micro	^___^	^___^	^___^	19	AB794387, AB794388

^a^Nucleotide sequences of two accession numbers were determined separately by forward and reverse primers in one clone.

^b^The cDNA fragment were obtain from *G*. *hokouensis*, which were mapped in our previous study (Kawai et al. [39]). For mapping of *ATP5A1*, *ACO1/IREBP* and *CHD1*, total length of cDNA fragment concatenated with multiple

–: No data

Dual-color FISH was performed to compare the chromosomal locations of the 5S rRNA genes with those of the 18S–28S rRNA genes and telomeric (TTAGGG)n sequences. We labeled 250 ng of the 5S rDNA probe with digoxigenin-11-dUTP (Roche Diagnostics) and hybridized it to *G*. *hokouensis* chromosomes with biotin-labeled 18S–28S rDNA probe or biotin-labeled 42-bp TTAGGG repeat. After hybridization, the digoxigenin- and biotin-labeled probes were stained with anti-digoxigenin-rhodamine Fab fragments (Roche Diagnostics) and avidin labeled with fluorescein isothiocyanate (avidin-FITC; Vector Laboratories), respectively.

## Results

### Karyotype and chromosomal locations of the 18S–28S and 5S rRNA genes and (TTAGGG)n sequences

**Fig 1:**
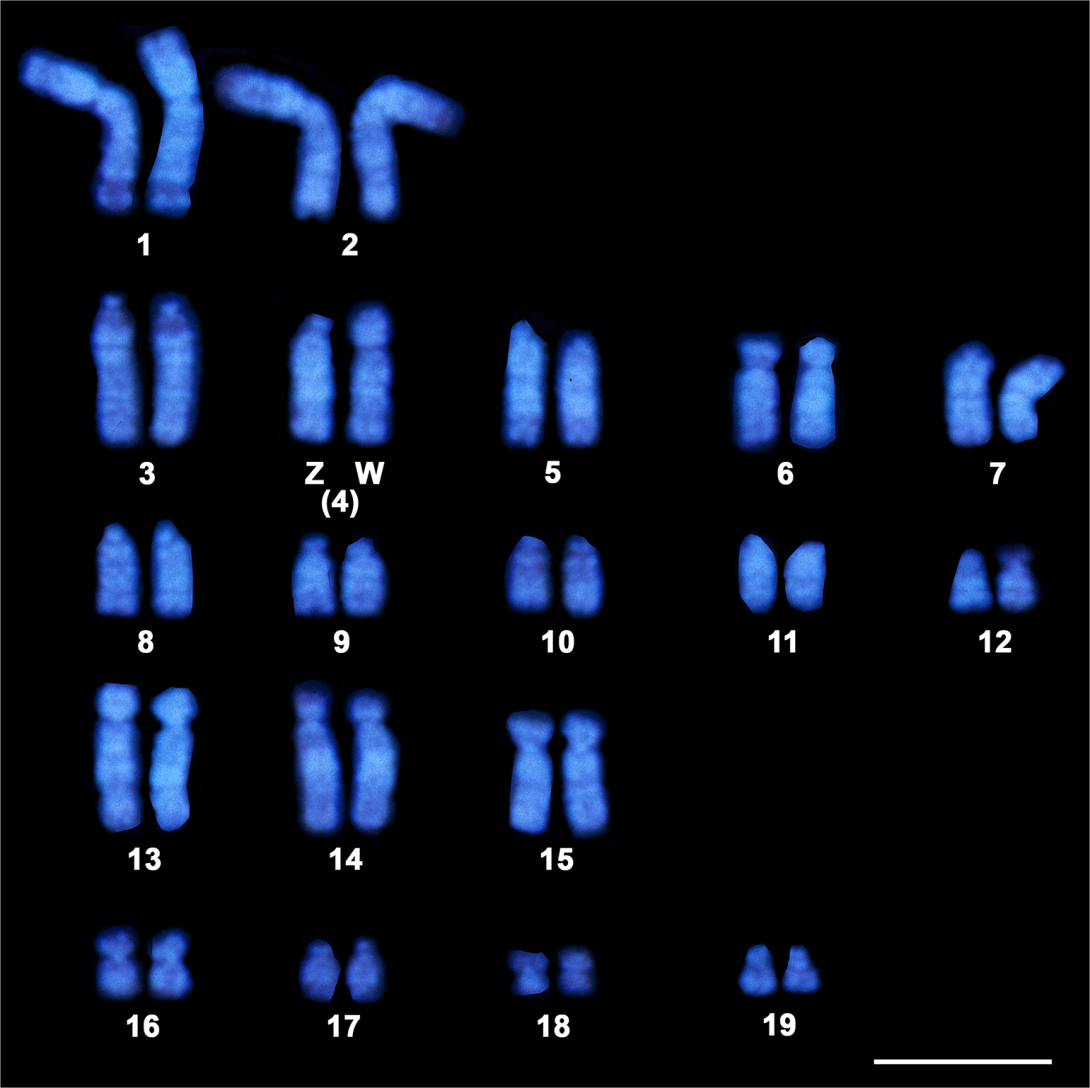
Hoechst 33258-stained karyotype of female *Gekko hokouensis*. Scale bar represents 10 μm.

Karyotyping of Hoechst 33258-stained metaphase spreads of female *G*. *hokouensis* showed a chromosome number of 2n = 38, which consisted of two pairs of large submetacentric chromosomes (1 and 2), eight pairs of large and/or medium-sized acrocentric chromosomes (3, 5, and 7–12), four pairs of large subtelocentric chromosomes (6 and 13–15), two pairs of small submetacentric chromosomes (16 and 19), one small subtelocentric chromosome pair (17), one small metacentric chromosome pair (18), and the heteromorphic Z and W sex chromosomes: the acrocentric Z chromosome and subtelocentric W chromosomes (Fig 1). The chromosomes were arranged according to the method described by Shibaike et al. [36] and Kawai et al. [39].

**Fig 2:**
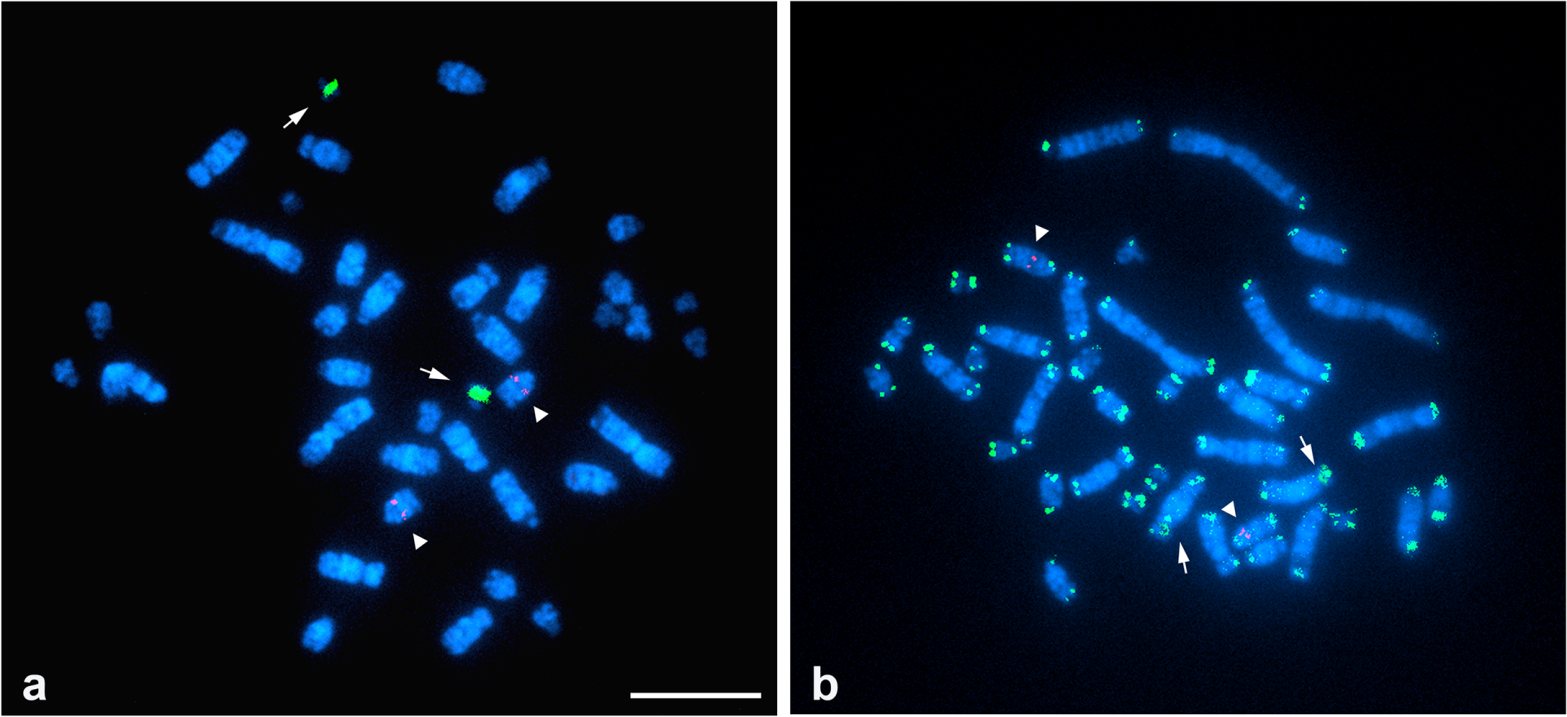
Chromosomal locations of the 18S–28S and 5S rRNA genes and (TTAGGG)n sequences in female *Gekko hokouensis*. (**a**) Hybridization pattern of FITC-labeled 18S–28S rRNA genes (green) and rhodamine-labeled 5S rRNA genes (red). Arrows indicate FISH signals of the 18S–28S rRNA genes, and arrowheads indicate signals of the 5S rRNA genes. (**b**) Hybridization pattern of FITC-labeled TTAGGG repeats (green) and rhodamine-labeled 5S rRNA genes (red). Arrows indicate signals of interstitial telomeric sites, and arrowheads indicate signals of the 5S rRNA genes. Scale bars represent 10 μm.

Fluorescence hybridization signals for the 18S–28S and 5S rRNA genes were localized to the pericentromeric region of chromosome 19 and proximal region of acrocentric chromosome 8, respectively (Fig 2A). Hybridization signals of TTAGGG repeats were observed at telomeric ends of all chromosomes. An interstitial telomeric site (ITS) was found at the pericentromeric region of the long arm of chromosome 14 (Fig 2B).

### Chromosome homology between *G*. *hokouensis* and the chicken

**Fig 3:**
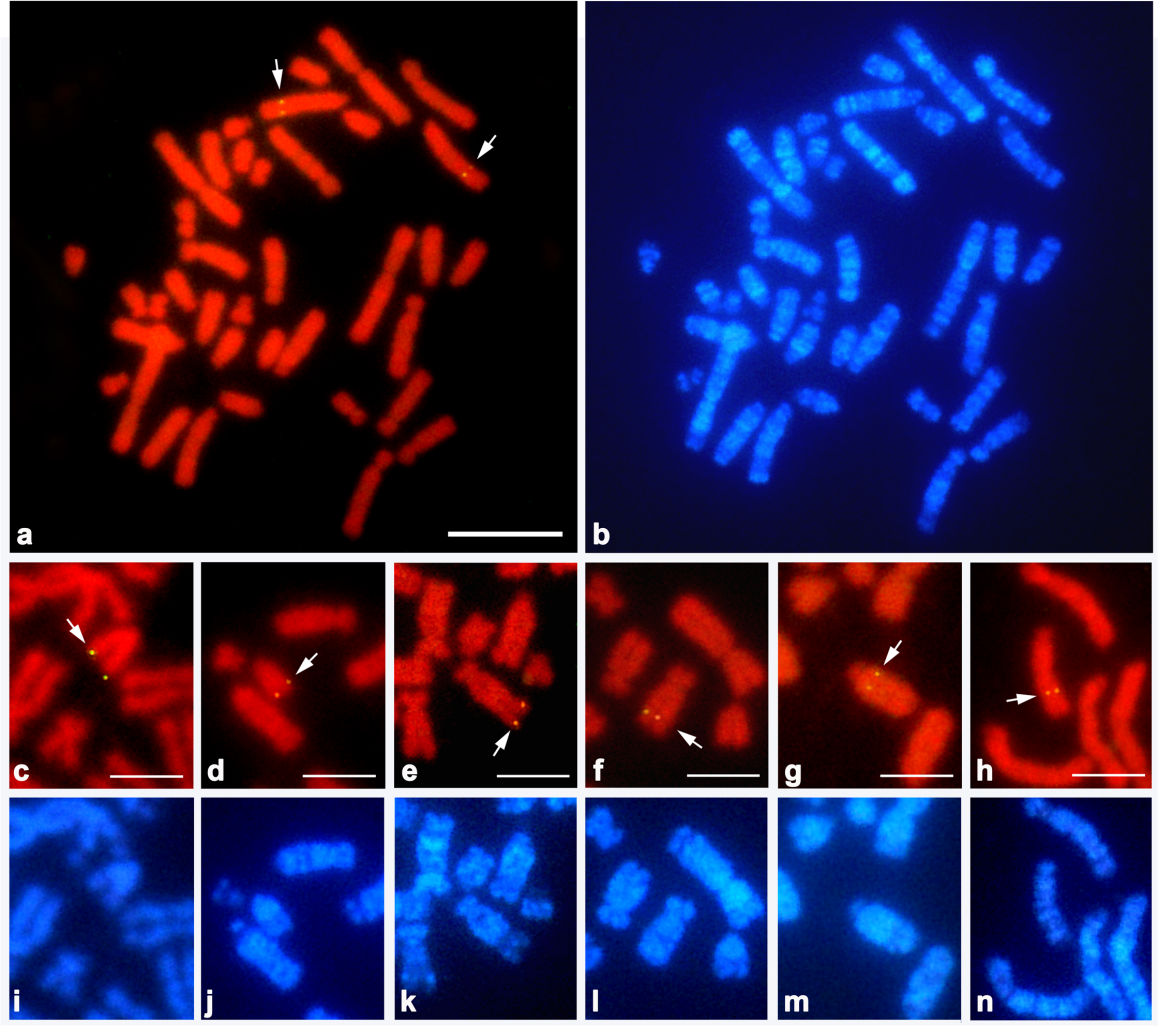
Chromosomal locations of cDNA fragments of functional genes in female *Gekko hokouensis*. *RBM12* was localized to chromosome 3 (GHO3) (**a**), *ATP2A2* to GHO11 (**c**), *SBNO1* to GHO11 (**d**), *SOX5* to GHO14 (**e**), *TLOC1* to GHO15 (**f**), *TMEM57* to GHO5 (**g**), and *WAC* to GHO8 (**h**). (**b, I, j, k, l, m, and n**) Hoechst 33258-stained patterns of the PI-stained metaphase spreads are shown in (**a, c, d, e, f, g,** and **h**). Arrows indicate the hybridization signals. Scale bars indicate 10 μm for (**a, b**) and 5 μm for (**c**–**h**).

**Fig 4:**
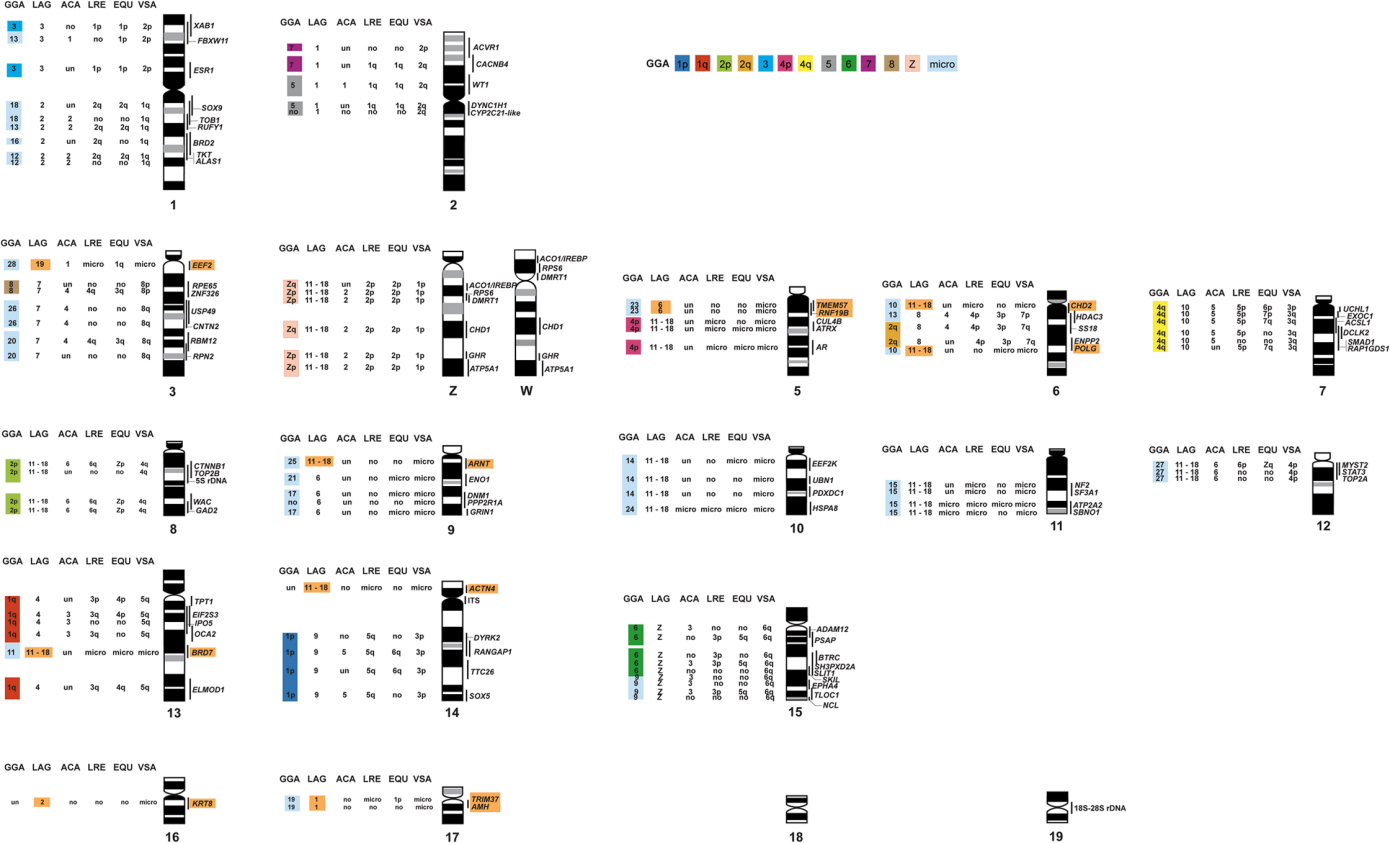
Cytogenetic map of *Gekko hokouensis*, which shows chromosome homologies with the chicken and five squamate reptiles. This map was constructed with 86 functional genes and 18S–28S and 5S rRNA genes. Chromosomal locations of *ATP5A1*, *GHR*, *CHD1*, *DMRT1*, *RPS6*, and *ACO1*/*IREBP* were obtained from Kawai et al. [39]. The idiogram of *G*. *hokouensis* chromosomes was constructed according to Hoechst 33258-stained band patterns. Locations of the genes on *G*. *hokouensis* chromosomes are shown to the right of the chromosomes. The chromosome numbers show the chromosomes of the chicken (*Gallus gallus*, GGA), green anole (*Anolis carolinensis*, ACA), butterfly lizard (*Leiolepis reevesii rubritaeniata*, LRE), Japanese four-striped rat snake (*Elaphe quadrivirgata*, EQU), water monitor lizard (*Varanus salvator macromaculatus*, VSA), and sand lizard (*Lacerta agilis*, LAG), which show homologies with *G*. *hokouensis* chromosomes. no, no data on chromosome homology; un, a gene whose chromosomal location remains undetermined. Orange highlight indicates the genes that are homologous to chromosome segments of LAG. These genes are located on LRE, EQU, or VSA microchromosomes. The chromosomal locations of genes in the squamate reptiles were obtained from the following sources: *L*. *reevesii rubritaeniata* from Srikulnath et al. [11], *A*. *carolinensis* from Alföldi et al. [14], *E*. *quadrivirgata* from Matsubara et al. [9, 10], *V*. *salvator macromaculatus* from Srikulnath et al. [12], and *L*. *agilis* from Srikulnath et al. [13].

**Fig 5:**
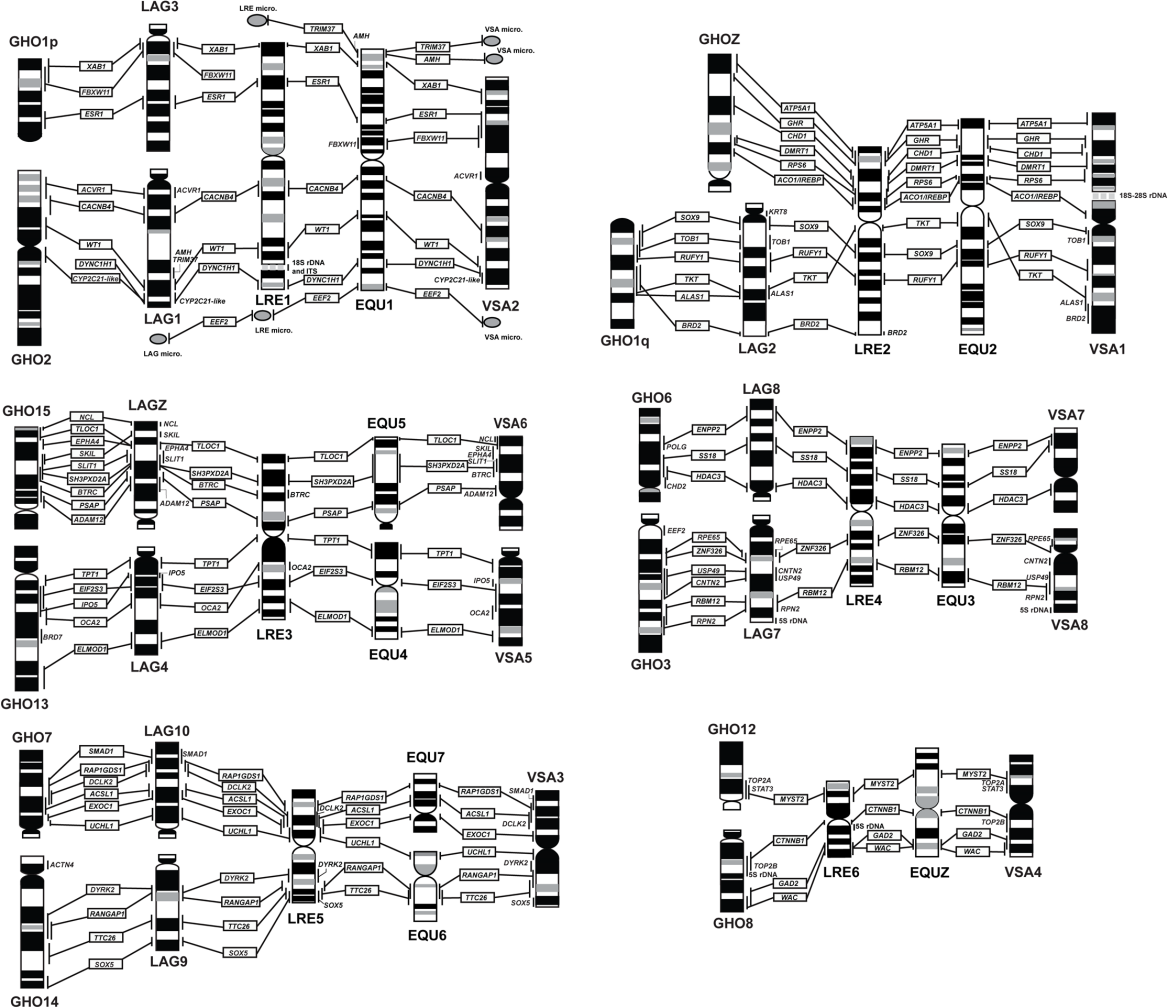
Comparative cytogenetic maps of macrochromosomes among *Gekko hokouensis*, *Lacerta agilis*, *Varanus salvator macromaculatus*, *Leiolepis reevesii rubritaeniata*, and *Elaphe quadrivirgata*, which were constructed with 68 functional genes. The chromosome map of *L*. *reevesii rubritaeniata* (LRE) was obtained from Srikulnath et al. [11]. The idiogram of *E*. *quadrivirgata* (EQU) macrochromosomes was obtained from Matsuda et al. [8] and chromosomal locations of the genes in *E*. *quadrivirgata*, from Matsubara et al. [9, 10]. The chromosome map of *V*. *salvator macromaculatus* (VSA) and *L*. *agilis* (LAG) were obtained from Srikulnath et al. [12, 13]. *G*. *hokouensis* chromosomes GHOZ, GHO6, GHO7, GHO12, and GHO15 and LAGZ, LAG8, LAG10, VSA3, VSA6, VSA7, EQU5, and EQU7 are inverted to facilitate comparison.

Eighty genes were newly mapped to *G*. *hokouensis* chromosomes in the present study, in addition to six Z-linked genes (*ATP5A1*, *GHR*, *CHD1*, *DMRT1*, *RPS6*, and *ACO1*/*IREBP*) that were mapped in our previous study [39]. We constructed a cytogenetic map for *G*. *hokouensis* with 86 functional genes (Figs 3–5), and to the best of our knowledge, this is the first comprehensive cytogenetic map for gekkotan lizards. More than 40 metaphase spreads were observed for each gene, with hybridization efficiencies ranging from approximately 30% to 80%. Chromosome homology between *G*. *hokouensis* and the chicken was analyzed using the chicken genome database (http://www.ncbi.nlm.nih.gov/genome/guide/chicken/). Nine genes that were mapped to *G*. *hokouensis* chromosome (GHO) 1 were localized to chicken (*Gallus gallus*) chromosomes (GGA) 3, 12, 13, 16, and 18 (Table 1, Figs 3 & 4). Five genes mapped to GHO2 were localized to GGA5 and GGA7. Seven genes on GHO3 were located on GGA8, GGA20, GGA26, and GGA28. GHO4 (the Z sex chromosome) corresponded to GGAZ, and GHO5 showed homology with GGA4p and GGA23. Five genes on GHO6 were localized to GGA2q, GGA10, and GGA13, and six genes on GHO7 were localized to GGA4q. GHO8 was homologous to GGA2p (Figs 3 & 4). GHO9 showed homology with GGA17, GGA21, and GGA25; GHO10, with GGA14 and GGA24; GHO11, with GGA15; and GHO12, with GGA27. Six genes on GHO13 were located on GGA1q and GGA11. GHO14 was homologous to GGA1p; and GHO15, to GGA6 and GGA9 (Figs 3 & 4). The chromosomal location of *KRT8*, which has not been determined in the chicken, was mapped to GHO16, and *TRIM37* and *AMH* located on GHO17 were localized to GGA19. No functional genes were mapped to GHO18 and GHO19 in the present study.

## Discussion

### Karyotype and chromosomal distribution of rRNA gene clusters in Gekkonidae

The karyotype of *G*. *hokouensis* (2n = 38) is composed of chromosomes in gradually decreasing size including several small pairs but without dot-shaped microchromosomes [36, 39]. Such an arrangement is commonly observed in gekkonid karyotypes [1]. The common diploid chromosome number of most *Gekko* species is 38 (FN = 42), which is slightly less than that of *Hemidactylus* species (2n = 40–46, FN = 40–46). Comparative chromosome painting for seven *Gekko* and *Hemidactylus* species revealed that the linkage groups of chromosomes have been highly conserved within each genus and between two genera [1, 42]. This finding suggests that the variation in chromosome number between *Gekko* and *Hemidactylus* is mainly caused by centric fusion and/or fission of several chromosome pairs.

In this study, 18S–28S rRNA genes were localized to the pericentromeric region of GHO19. A similar result was found for four other *Gekko* species, namely, *Gekko shibatai*, *G*. *tawaensis*, *G*. *yakuensis*, and *G*. *vertebralis*, and 11 *Paroedura* species of Gekkonidae, in which the Ag-NOR staining region is localized to the smallest chromosome pairs [36, 43]. However, the chromosomal locations of 18S–28S rDNA vary in other gekkotan taxa—on a pair of large or medium-sized chromosomes in *Hemidactylus platyurus*, *Ebenavia inunguis*, and *Uroplatus phantasticus* of Gekkonidae [42, 43], and *Gymnodactylus amarali* and *G*. *darwinii* of Phyllodactylidae [44]; and on X_1_ chromosome in *Coleonyx elegans* of Eublepharidae [37]. These results suggest that the chromosomal locations of the 18S–28S rRNA genes are diverse in Gekkota.

Chromosomal locations of the 5S rRNA genes have not been reported for Gekkota. In this study, the 5S rRNA genes were localized to the proximal region of GHO8. The genes are located on chromosome 6q of *L*. *reevesii rubritaeniata*, which has homology with GHO8 [11, 41], but on chromosome 8q of *V*. *salvator macromaculatus* and chromosome 7 of *L*. *agilis*, both of which are homologous to GHO3 [12, 13]. These results suggest that the chromosomal locations of the 5S rRNA genes are also diverse in squamate reptiles.

### Reorganization of macrochromosomes in Gekkota

Comparison of the cytogenetic map for *G*. *hokouensis* with those of the chicken and four episquamate reptiles (*L*. *agilis*, *E*. *quadrivirgata*, *V*. *salvator macromaculatus*, and *L*. *reevesii rubritaeniata*) revealed that 11 chicken macrochromosomes and/or macrochromosome arms (GGA1p, GGA1q, GGA2p, GGA2q, GGA3, GGA4q, GGA5, GGA6, GGA7, GGA8, and GGAZ), which showed homologies with most of the macrochromosomes of the three Toxicofera species and eight macrochromosomes of *L*. *agilis*, were highly conserved in 10 chromosomes of *G*. *hokouensis* [GHO1p, GHO2, GHO3, GHOZ(4), GHO6, GHO7, GHO8, GHO13, GHO14, and GHO15] (Table 1, Figs 3–5). These results collectively suggest that the linkage groups of the chicken, Toxicofera, and Lacertidae are also highly conserved in *G*. *hokouensis*, although *G*. *hokouensis*, as well as *L*. *agilis*, has a diversified karyotype. Therefore, comparative cytogenetic maps of the representatives of the three taxa (Toxicofera, Lacertidae, and Gekkota) enable us to delineate the process of karyotypic reorganization in squamate reptiles based on the most parsimonious explanation for chromosomal rearrangements.

**Fig 6:**
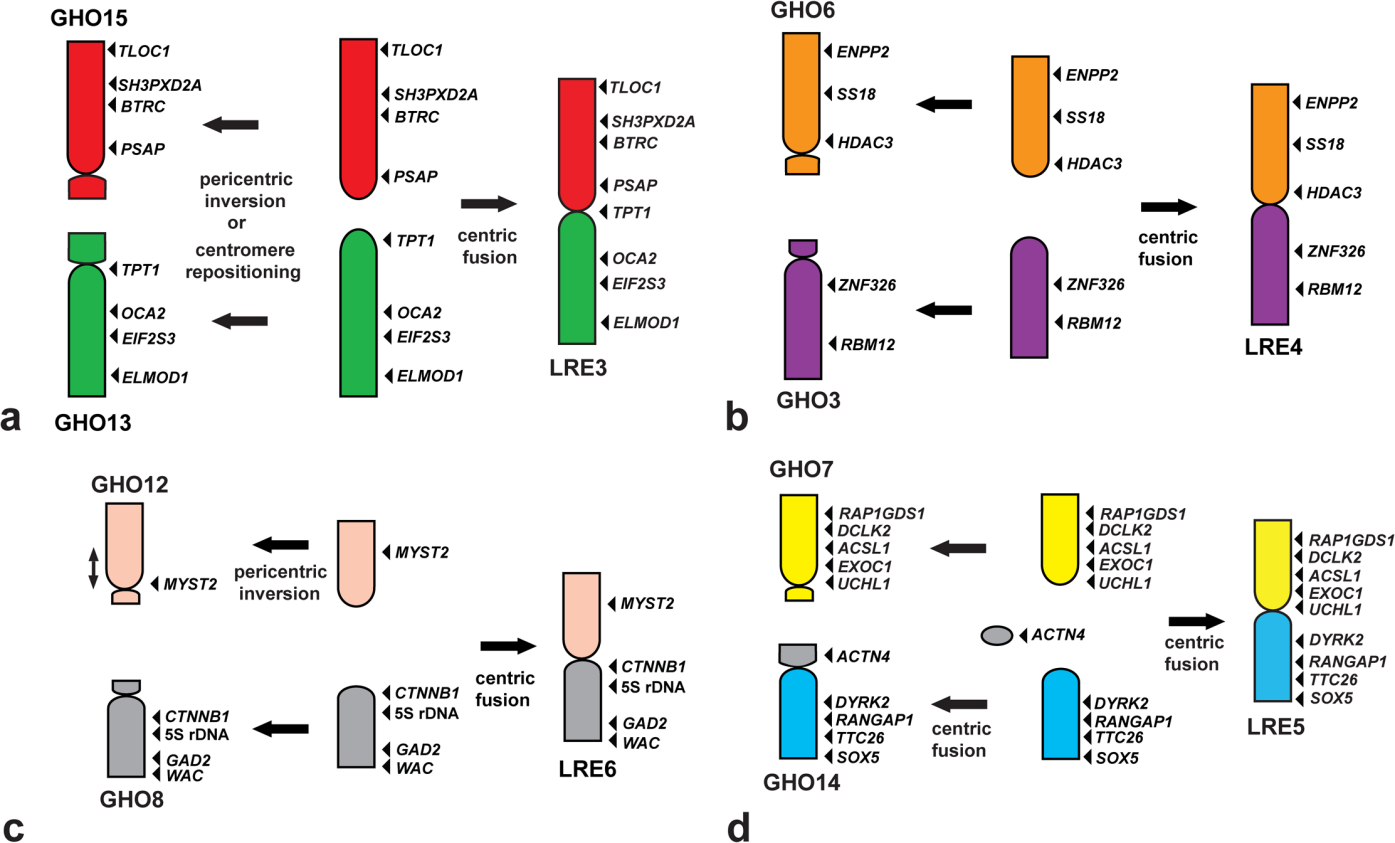
Schematic representation for the process of chromosomal rearrangements that occurred among *Gekko hokouensis* chromosomes (GHO) 3, 6–8, and 12–15 and *Leiolepis reevesii rubritaeniata* chromosomes (LRE) 3–6. The diagram schematically summarizes the chromosomal rearrangements that occurred in LRE3, GHO13, and GHO15 (**a**); LRE4, GHO3, and GHO6 (**b**); LRE6, GHO8, and GHO12 (**c**); and LRE5, GHO7, and GHO14 (**d**). GHO6, GHO7, GHO12, and GHO15 are inverted to facilitate comparison. Chromosomal locations of the genes are shown to the right of the chromosomes by using arrowheads. Homologous chromosomes and/or chromosome segments are shown using the same color. Arrows indicate the directions of the chromosomal rearrangements.

GHO13 corresponds to *L*. *reevesii rubritaeniata* chromosome (LRE) 3q (LRE3q), and GHO15 to LRE3p. This indicates the possibility that LRE3 resulted from centric fusion of the acrocentric proto-GHO13 and proto-GHO15 (Fig 6A) and that the present subtelocentric GHO13 and GHO15 are derivatives of the ancestral type of acrocentric chromosomes homologous to *L*. *agilis* chromosome (LAG) 4 (LAG4) and LAGZ(5) (Figs 5&6A). Alternatively, the present form of GHO13 and GHO15 can be explained by centric fission of an ancestral bi-armed chromosome homologous to LRE3, followed by pericentric inversion or centromere repositioning. However, a centric fusion event is more likely, considering that Gekkota is phylogenetically located at the basal position and that *Lacerta* is positioned in a lineage different from Toxicofera [6].

GHO3 and GHO6 are homologous to LRE4q and LRE4p, respectively. GHO8 corresponds to LRE6q and GHO12, to LRE6p. These four GHO chromosomes may have been derived from centric fission of the ancestral bi-armed macrochromosomes or the bi-armed chromosomes may have resulted from centric fusion of the ancestral types of acrocentric chromosomes (Figs 6B&6C). However, centric fusion is most likely because GHO3, GHO6, GHO8, and GHO12 are considered the prototypes in the lineages of squamate reptiles, according to the phylogenetic relationship in which Gekkota are located near the basal position [6].

FISH analysis with telomeric TTAGGG repeats successfully detected an ITS in the pericentromeric region of GHO14, which is considered to be a relic of tandem fusion of chromosomes [41, 45, 46] (Figs 2&4); this suggests the occurrence of chromosome fusion between a microchromosome (GHO14p) and the acrocentric proto-GHO14q in *G*. *hokouensis*. The bi-armed LRE5 may have resulted from centric fusion between the acrocentric proto-GHO14q and proto-GHO7 (Fig 6D).

**Fig 7:**
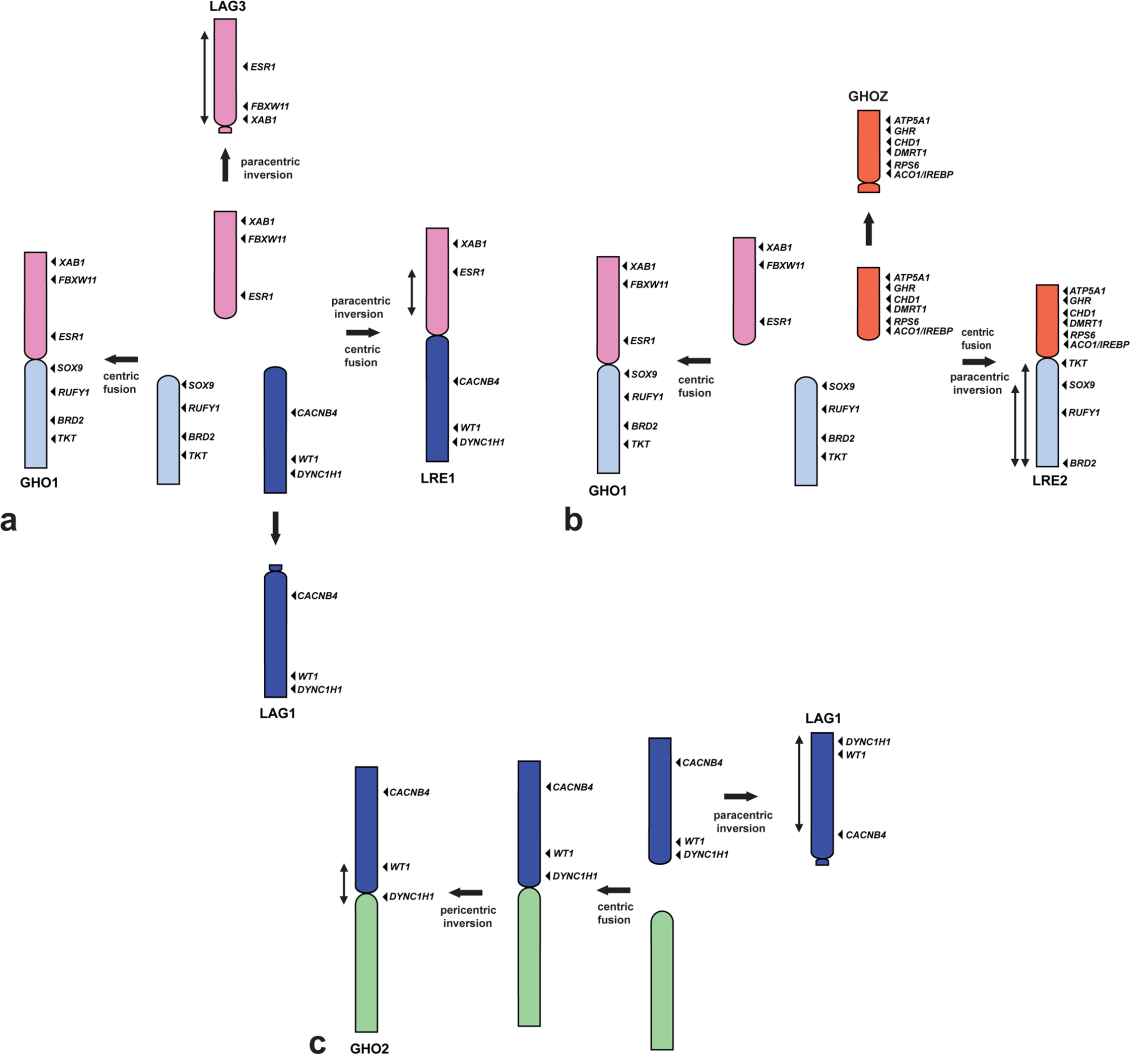
Schematic representation for the process of chromosomal rearrangements that occurred among *Gekko hokouensis* chromosomes (GHO) 1, 2, and Z, *Lacerta agilis* chromosomes (LAG) 1 and 3, and *Leiolepis reevesii rubritaeniata* chromosomes (LRE) 1 and 2. The diagram schematically summarizes the occurrences of LRE1, GHO1, LAG1, and LAG3 (**a**); LRE2, GHO1, and GHOZ (**b**); and LAG1 and GHO2 (**c**). LAG3 in (**a**) and GHOZ in (**b**) are inverted to facilitate comparison. Chromosomal locations of the genes are shown to the right of the chromosomes by using arrowheads. Homologous chromosomes and/or chromosome segments are shown using the same color. Arrows indicate the directions of the chromosomal rearrangements.

However, two of the largest chromosome pairs (GHO1 and GHO2) are bi-armed chromosomes. GHO1p is homologous to LRE1p and LAG3; however, the gene order of GHO1p is different from that of LRE1p and LAG3. Considering the phylogenetic positions of Gekkota and *Lacerta*, it is most likely that LRE1 resulted from centric fusion between LAG1 and the acrocentric proto-GHO1p, followed by paracentric inversion in LRE1p. Proto-GHO1p may have fused with the acrocentric proto-GHO1q in *G*. *hokouensis*, leading to the present bi-armed GHO1. LAG3 may have been derived from proto-GHO1p by a large paracentric inversion (Fig 7A). GHOZ is homologous to LRE2p and GHO1q, to LRE2q (Fig 7B). LRE2 was probably derived from centric fusion between the acrocentric proto-GHOZ and proto-GHO1q, followed by paracentric inversions in LRE2q, based on the evidence of chromosome homology with the other three Toxicofera species (Fig 5) and their phylogenetic relationships [6].

LRE1p is homologous to GGA3 and LRE1q, to GGA5 and GGA7. LRE2p is homologous to GGAZ (Table 1 and Fig 4). Pokorná et al. [47, 48] conducted chromosome painting of 13 squamate reptiles that are grouped into Episquamata and Scincoidea clades, which are phylogenetically distinct from Gekkota, by using GGA3, GGA5, GGA7, and GGAZ probes. The results showed that a short arm of a bi-armed macrochromosome pair was painted with GGA3 and a long arm with GGA5 and GGA7 in most species. GGAZ was homologous to either the short arm of the bi-armed macrochromosome pair or acrocentric chromosomes. These results suggest that LRE1, LRE2, and GHO1 are derived from centric fusion between the ancestral types of acrocentric chromosomes.

GHO2 is also supposed to have occurred by centric fusion of the acrocentric proto-GHO2q and proto-LAG1, followed by a small pericentric inversion (Fig 7C); however, chromosomal rearrangements that occurred on GHO2q could not be estimated precisely because no GHO2q homologs have been mapped to chromosomes of other species. LAG1 may have been obtained from proto-LAG1 by paracentric inversion.

Karyotype data for Gekkota suggest that a typical gekkonid karyotype is composed of a graded series of acrocentric chromosomes with few or no bi-armed chromosomes and no distinct boundary between macrochromosomes and microchromosomes. The 2n = 38 acrocentric karyotype is considered to be the ancestral karyotype common in Gekkonidae [36, 38], Diplodactylidae [34, 35] and Eublepharidae [37], except Phyllodactylidae (2n = 32–44) [44, 49], Shaerodactylidae (2n = 32–44) [50, 51]. Changes in chromosome numbers and fundamental numbers are predominantly reflected by fusions and fissions, and pericentric inversions and/or centromere repositioning, respectively [42, 52, 53]. The results of the present study suggest that the acrocentric macrochromosomes of *G*. *hokouensis* retain the ancestral type of Gekkota chromosomes and that most of the bi-armed chromosomes in Episquamata may have been formed by centric fusion of the ancestral acrocentric chromosomes, which had been contained in the ancestral karyotype of Gekkota (Figs 6&7).

### Reorganization of microchromosomes in Gekkota

Considering that karyotypes with many microchromosomes are found in majority of the squamate reptiles, it is most likely that the ancestral karyotype of squamate reptiles was composed of both macrochromosomes and microchromosomes [1, 15], and that all or most of the microchromosomes were lost in the lineages of Gekkota and Lacertidae. Four pairs of *G*. *hokouensis* chromosomes (GHO5, GHO9, GHO10, and GHO11) and one pair of *L*. *agilis* chromosomes (LAG6) are composed of tandem fused-chromosome segments that have homologies with microchromosomes of *L*. *reevesii rubritaeniata*, *E*. *quadrivirgata*, and *V*. *salvator macromaculatus* [13, in this study]. Insertions or fusions of microchromosome segments were also found in GHO3, GHO6, GHO13 and GHO14, and LAG1 and LAG2 (Table 1, Figs 4&5) [13]. These results collectively suggest that the disappearance of microchromosomes in *G*. *hokouensis* and *L*. *agilis* was due to repeated fusions between microchromosomes and/or macrochromosomes and microchromosomes that existed in the ancestral karyotype of squamate reptiles. This process probably occurred independently in each lineage of Gekkota and Lacertidae because no homology was found in the chromosomes, which resulted from insertions or fusions of microchromosomes, between the two lineages. However, comparative gene mapping for species that are closely related to Gekkota or Lacertoidea, such as Dibamidae with few or no microchromosomes (as is obvious from Fig 1 of Cole and Gans [54]) or Gymnophthalmidae and Amphisbaenia with many microchromosomes [1, 29, 55–57], and far-related Sphenodontidae species with several pairs of microchromosomes [58] is required to discuss karyotype evolution in squamate reptiles in more detail.

In this study, a comparison of the cytogenetic maps of six squamate reptiles (*G*. *hokouensis*, *L*. *agilis*, *E*. *quadrivirgata*, *V*. *salvator macromaculatus*, *L*. *reevesii rubritaeniata*, and *A*. *carolinensis*) enabled us to delineate the process of chromosomal reorganization in Gekkota, Lacertidae, and Toxicofera. These cytogenetic data would also be an essential prerequisite for the future genome projects of squamate reptiles, for example, *de novo* sequence assembly after whole-genome sequencing by using next-generation sequencing technology. These data will provide insight into the phylogenetic hierarchy of genome evolution in squamate reptiles.

## Data Availability

All relevant data are within the paper.
